# Editorial: Improving Wellbeing in Patients With Chronic Conditions: Theory, Evidence, and Opportunities

**DOI:** 10.3389/fpsyg.2022.868810

**Published:** 2022-03-14

**Authors:** Andrew H. Kemp, Jeremy Tree, Fergus Gracey, Zoe Fisher

**Affiliations:** ^1^School of Psychology, Faculty of Medicine, Health & Life Science, Swansea University, Swansea, United Kingdom; ^2^Regional Neuropsychology and Community Brain Injury Service, Morriston Hospital, Swansea, United Kingdom; ^3^Department of Clinical Psychology and Psychological Therapies, Norwich Medical School, Univeristy of East Anglia, Norwich, United Kingdom; ^4^Health and Wellbeing Academy, Faculty of Medicine, Health and Life Science, Swansea University, Swansea, United Kingdom

**Keywords:** wellbeing science, vagus nerve, individual wellbeing, collective wellbeing, community, chronic conditions, healthcare

The global epidemiological transition characterizes a shift in the nature of health and disease from acute disease to chronic conditions. Chronic conditions have now superseded acute conditions as leading burdens of morbidity, mortality, and health care expenditures (Murray and Lopez, 1997a,b; Ferrari et al., 2014). In fact, 80.45% of years lived with disability (YLDs) are attributable to chronic conditions, including back pain, depressive, and headache disorders (http://ihmeuw.org/5nrp). Furthermore, despite an increasing lifespan, we are living with more disease and infirmity (Vos et al., 2015; Kyu et al., 2018). Despite this transition our models of health care have not adapted to reflect these changes (Murray and Lopez, 1997b). Accordingly, there is an urgent need to develop more effective approaches to managing chronic conditions both to enhance care and to address the burden chronic conditions are posing on healthcare systems. It is timely then to discuss the theory, evidence, and opportunities for building wellbeing in the increasing number of people who are living with conditions. Conditions that must be managed and for which “cure” is seldom possible.

## Theoretically Informed Holistic Interventions are Needed

Despite being designed to treat acute illness and disease, “the acute medical model” has become the dominant model of healthcare. We have argued that this model of healthcare constrains opportunities to develop evidenced based holistic approaches for supporting people living with chronic conditions (Fisher et al., 2022; Kemp and Fisher, 2022). There is a pressing need for approaches that consider all domains of functioning (biological, cognitive, emotional, social) and their interactions as well as considering the influence of wider systemic factors such as our relationships, community, environment and socio-contextual factors (Wilson et al., 2009; Gracey et al., 2015; Mead et al., 2019, 2021; Wilkie et al., 2021; Fisher et al., 2022). This point is illustrated by several studies of disease burden included in our Research Topic (Belrose et al.; Barreira et al.; Biehl et al.; Huber and Havas; Bossy et al.).

For instance, Barreira et al. describe the profound impact of hepatitis C virus (HCV) on health, cognition and psychological wellbeing. The authors highlight the need for theoretically informed holistic interventions that address the physical, cognitive, psychological and social sequelae of the disease in order to provide more effective outcomes. Similarly, a study by Biehl et al. explored the relationship between personality, perceived stress and tinnitus related distress. The authors reported significant differences on a variety of personality indices compared to the general population, and that tinnitus-related distress is mediated by differential interactions between personal factors and perceived stress. The role of emotion-focused treatment strategies are emphasized including for example, compassion focused therapy or schema therapy. Another study by Huber and Havas points out that although cochlear implants have been shown to support language development in hearing-impaired children, speech recognition in noisy environments remains limited. This has implications for the quality of life of children and adolescents with cochlear implants highlighting a need for broader solutions including psychological support and environmental modification. Finally, the paper by Bossy et al. emphasize a need to compare different psychological treatment options for patients diagnosed with schizophrenic psychosis in an inpatient settings. The authors present a protocol for a randomized controlled trial of a novel treatment intervention to improve neuro- and social-cognition as well as emotion regulation skills.

Chronic conditions are often associated with comorbidity and “multimorbidities” (Harrison et al., 2021). Multimorbility has been identified as a priority for research (Mangin et al., 2018) and health care systems (Whitty et al., 2020) because it is highly prevalent occurring in a third of adults living in the community (Nguyen et al., 2019) and differentially affecting those from the most socioeconomically disadvantaged communities (McLean et al., 2014). By means of example, two papers in our Research Topic report on the prevalence of psychiatric comorbidity alongside other chronic conditions. In a paper by Alsaadi et al., psychiatric comorbidity in neurological disorders is reported to be as high as 39% for symptoms of depression and 34% for anxiety. A related paper by Almeida et al. reports on the prevalence of major depressive disorder in patients with variety of chronic diseases including diabetes, rheumatoid arthritis, cancer and Parkinson's disease. The authors point out that the prevalence of major depressive disorder is two- to three-fold higher in patients with chronic diseases than in the general population. Consistent with these reports, a recent meta-analysis reported a pooled prevalence of psychiatric disorders in patients with chronic physical diseases of 36.6% (95% CI 31.4–42.1) and a pooled odds ratio of 3.1 (95% CI, 1.7–5.2; Daré et al., 2019). A critical issue here is that psychological distress is associated with premature mortality in a dose response relationship (Russ et al., 2012) and that psychiatric disorders, which are themselves chronic conditions, have the potential to reduce life expectancy, with impacts that are equivalent to, and sometimes exceed, the effects of heavy smoking (Chesney et al., 2014). These findings are in keeping with many robust epidemiological studies showing that physical diseases and common mental health conditions are strongly inter-connected, highly co-morbid and share critical pathways to ill health and disease (Verhaak et al., 2005; Druss and Walker, 2011; O'Neil et al., 2015; Dai et al., 2020). This is not surprising given compelling evidence of a tight coupling between pathways subserving both physical and mental health (Thayer et al., 2009; Kemp et al., 2017b).

## Reducing Illbeing Is not the Same as Promoting Wellbeing

It has become commonplace for the construct of wellbeing to be interpreted as synonymous with the “absence of distress” rather than being associated with the presence of key determinants of wellbeing. Similarly, health has also been viewed as the absence of illness or disease. Such ideas have been enshrined in models of healthcare which are often centered around reducing impairment and distress. Again, this bias misses opportunities to promote acceptance of difficult emotions and life experiences that cannot be changed and this is the crux of the experience of those who must live with a chronic condition(s). It also neglects the growing evidence highlighting the role of promoting positive emotion to facilitate creative, flexible, and novel ways of thinking and behaving, that builds lasting physical, intellectual, psychological, and social resources (Fredrickson, 1998; Boehm and Kubzansky, 2012; Lee et al., 2019; Rozanski et al., 2019). Moreover, positive emotions and wellbeing have important implications for morbidity from a host of conditions and disease as well as premature mortality (DuBois et al., 2012; Steptoe et al., 2015). Accordingly, our Research Topic includes papers that are consistent with positive psychology and third wave CBT (e.g., mindfulness). Here the emphasis is on changing one's relationship with difficult thoughts (Robinson et al., 2019) thereby promoting acceptance and emotional balance as well as building positive emotions. For example, through connection with the present moment as well as through practicing exercises and activities that promote positive emotion (e.g., Tulip et al.; Di Giacomo et al.; Rowlands et al.; Marks et al.; Rodrigues et al.; Wigham et al.). These studies report on the potential for improvements in a range of wellbeing-related variables including connection, kindness, compassion, equanimity, quality of life, coping behaviors, self-concept, and positive mood. Taken together, this work shows the potential for promoting wellbeing in people living with chronic conditions.

## Theoretical Foundations

Our Research Topic includes several papers that explore the theoretical foundations for promoting wellbeing in people living with chronic conditions, shedding light on the potentially contradictory idea that people living in ill-health also have capacity for wellbeing. The unique perspective article by Hunter presents a clinician and service users' perspective on managing multiple sclerosis based on the P-P-P [Pleasure-Purpose-Practice] framework, encompassing core principles from positive psychology and behavior change science. Hunter emphasizes that it is indeed possible to experience “pleasure” despite pain or disability, highlighting that the experience of positive and negative emotions is not mutually exclusive. In another original research article, Roşca et al. report on the experiences of patients who had suffered limb amputation, observing that despite the experience of negative emotions (e.g., anxiety, guilt, and anger), social isolation and constraints on roles and routines, participants also presented with hope and determination to prevail, when supported by family, friends, and colleagues.

These insights are consistent with theory. For instance, the two continua model (Westerhof and Keyes, 2010) presents mental illness and (positive) mental health as related but distinct phenomena, reinforcing the possibility that one can experience ill-health (including mental illness) *at the same time as* a moderate level of mental health. Intriguingly, two papers in our Research Topic sought to test this hypothesis. The first, included people with DSM-5 diagnoses related to paranoid thinking (including Schizophrenia, Brief Psychotic Disorder, Delusional Disorder, or Substance/Medication-Induced Psychotic Disorder) who were without mood symptoms (Asensio-Aguerri et al.). The second included people living with an eating disorder (de Vos et al.). Reported findings were consistent with the two-continua model including the observation that positive mental health and paranoid thinking lay on two—rather than one—unipolar dimensions, and that 13% of those with eating disorders reported high levels of wellbeing (flourishing). Our own study published in this Research Topic (Tulip et al.) reports that an 8-week positive psychotherapy intervention facilitated positive emotions, empowerment, skills, and social opportunity in people living with acquired brain injury. Together, these findings defy the stereotype that “people living with chronic conditions do not have capacity for wellbeing.” This work aligns closely with that of psychologist, Paul Wong, who emphasizes the important roles of meaning and purpose in life for experiencing wellbeing despite hardship and suffering, ideas that have been described as second wave positive psychology (Wong, 2019).

Other papers in our Research Topic also raise interesting considerations relating to identity (Kerr et al.), quality of life (Wigham et al.), and post-traumatic growth (Pérez-San-Gregorio et al.). For instance, the conceptual analysis by Kerr et al. describes a novel coaching-related intervention that is used to facilitate adaptive change with a focus on building skills to reconstruct narrative identity and foster hope. Often when one develops a chronic condition, the person will experience significant biopsychosocial disruption including pathology-related impact, impairments in cognition and emotions and reduced capacity to engage in activities that were previously enjoyed. This can lead to subjective sense of “threats to identity” (Gracey et al., 2019) profoundly linked to social and interpersonal contexts (Gracey et al., 2008; Gracey and Ownsworth, 2012). The Y-Shaped model of rehabilitation (Gracey et al., 2009) focuses intervention on the psychological and social discrepancies between the current and ideal “selves,” and moving forwards to identify and build on continuities in identity, as realized and experienced through the lived environment. A similar point is made in the narrative approach to identity adaptation following stroke or brain injury (Ellis-Hill et al., 2009; Whiffin et al., 2021). Moving beyond identity reconstruction, the article by Pérez-San-Gregorio et al. reports on the capacity for people undergoing liver transplants to experience post-traumatic growth, an experience that is motivated by personal distress and a need to regain intra-psychic balance.

## Opportunities to Promote Mental and Physical Health

Our own work (Kemp and Quintana, 2013; Kemp et al., 2017a; Mead et al., 2021) as well as others have emphasized the vagus nerve as a structural link between physical and mental health. The vagus connects the central nervous system to many different organs including heart, gut, liver, and lungs. Vagal afferent fibers represent the most important function of the vagus, sending information from the viscera to the brain (Breit et al., 2018), providing a key pathway through which positive health behaviors may beneficially impact on mind. Reflecting a growing interest in the potential benefits of mind-body interventions to promote wellbeing in people living with chronic conditions, our Research Topic included papers on yoga (Telles et al.), dohsa-hou body movement relaxation tasks (Haramaki et al.; Zhu et al.). In their review article, Tarsha et al. further describe how a variety of physical interventions including massage therapy, tai-chi and dance therapy alleviate depression, anxiety, and stress while facilitating pain reduction through bidirectional pathways, which may include the vagus. In their systematic review, Edwards and Pinna examine the associations between vagal nerve function, interoception, and emotional regulation, which have important implications for wellbeing in people living with chronic conditions. Vagal nerve functioning was associated with better emotion regulation and psychological flexibility, suggesting that various interventions known to impact on vagal function may help to reduce psychological distress and promote wellbeing in people living with chronic conditions. A variety of interventions have been shown to improve vagal function including meditation, relaxation, nutrition, exercise, social connection, and spending time in nature (Kok et al., 2013; Richardson et al., 2016; Laborde et al., 2018; Young and Benton, 2018). We have sought to synthesize these findings in our GENIAL model of wellbeing, in which we define wellbeing as connection to self, others and nature, supported by functioning of the vagus nerve, which appears to provide a psychophysiological resource for connection and the subsequent experience of wellbeing (Kemp et al., 2017a; Mead et al., 2021; Kemp and Fisher, 2022).

## Opportunities to Promote Social Wellbeing

The study by Zhang et al. investigated the relationships among social support, physical comorbidity, health literacy, and depression in patients with hypertension in China, reporting that physical comorbidity is positively associated with depression, while health literacy and social support were negatively associated. The authors suggested that health education programmes combined with community-based activities may help to reduce the experience of depression. Several other papers in this Research Topic focus on community-based interventions in order to promote wellbeing in people living with chronic conditions (Tong et al.; Faw et al.; Smith et al.). For instance, the paper by Smith et al. report on the impact of an intergenerational dementia education program, with qualitative interview revealing positive changes in children's empathy and improved community awareness. The authors further highlighted the need for support from school and community partners as key to the success of the program. Of the 12 older adults and four carers interviewed, the intergeneration program was associated with positive emotions as a result of interacting with the children and positive self-changes.

## Other Opportunities

Other papers in our Research Topic focused on how digital health technologies might be used to promote wellbeing in people who live with chronic conditions. The study by Wulfovich et al. for example, was an explorative mixed-method study of 200 chronically ill patients, documenting how mobile apps and wearables might be designed to better promote self-efficacy, especially in regards to activities relating to chronic disease management. These ideas are expanded on in the opinion article by Bedrov and Bulaj, who focus on opportunities to promote positive thinking, self-esteem, and empowerment using motivational quotes through digital health technologies. The authors suggest that different categories of motivational quotes may be tailored for specific causes of low self-esteem. For example, when low self-esteem arises as a function of negatively comparing oneself to others, one approach might be to design motivational quotes encouraging positive self-evaluation allowing the recognition of personal strengths and values. Another article by Gorini et al. discusses how patients might not only be empowered in disease management but also in the achievement of positive experiences and experiential growth to find innovative and personalized ways to improve care using mobile health technology. Another paper by Faw et al. explored the potential for virtual reality to be used as a salutogenic design intervention for 16 older adults including three participants with dementia and two spousal caregivers. Although in early development, the authors argue for potential application amongst those who struggle to access “in real life” events due to conditions such as dementia, highlighting opportunities to increase enjoyment and engagement.

Taken together this work touches upon the huge potential of digital technology solutions, along with other assistive technology, to have a positive impact on health and wellbeing for those living with chronic conditions. However, a meta-synthesis from our own group show that complex barriers exist to acquiring and using appropriate assistive technology for people with chronic conditions. Barriers relate to the devices themselves, the individual context, healthcare context and wider societal barriers such as stigma (Howard et al., 2020). Finding interventions to overcome such barriers, for example greater involvement of patients in the design and provision of assistive technology, will help provide more usable assistive technology solutions that assist in empowering the individual in better managing their own health, improving independence, enabling great social connections, and as such promoting wellbeing. It is important assistive technology is integrated as a tool alongside other interventions in seeking to impact on health and wellbeing and that people living with chronic conditions are involved in the design of such interventions where possible.

## Discussion and Conclusions

In summary, our Research Topic encompasses a total of 31 articles that together highlight the societal burden associated with a variety of chronic conditions, while also emphasizing theory and potential interventions for facilitating wellbeing in those who must live with such conditions. There is, across this work, a clear argument for an integrated, holistic approach to wellbeing within a biopsychosocial framework that is connected to the wider sociostructural and cultural context. From this body of work there is hope for radical developments in the application of technology, traditional cultural practices such as art, music, Tai Chi, and storytelling and psychological interventions to improve wellbeing in chronic conditions. It is now incumbent on us working in academia and the healthcare sector to realize the many opportunities for promoting wellbeing in chronic conditions, especially given the increasing available evidence emphasizing the bidirectional impacts between mental and physical health that may—if not addressed—contribute to years lived with disability and lives lost. We hope that this Research Topic will inspire more researchers to further develop the emerging evidence base that explores capacity of people with a chronic condition to experience wellbeing.

## Author Contributions

AK and ZF wrote the first draft of the editorial, which FG and JT subsequently refined for publication. All authors were Research Topic editors. All authors contributed to the article and approved the submitted version.

## Conflict of Interest

The authors declare that the research was conducted in the absence of any commercial or financial relationships that could be construed as a potential conflict of interest.

## Publisher's Note

All claims expressed in this article are solely those of the authors and do not necessarily represent those of their affiliated organizations, or those of the publisher, the editors and the reviewers. Any product that may be evaluated in this article, or claim that may be made by its manufacturer, is not guaranteed or endorsed by the publisher.
